# Unprecedented and highly stable lithium storage capacity of (001) faceted nanosheet-constructed hierarchically porous TiO_2_/rGO hybrid architecture for high-performance Li-ion batteries

**DOI:** 10.1093/nsr/nwaa028

**Published:** 2020-02-20

**Authors:** Wen-Bei Yu, Zhi-Yi Hu, Jun Jin, Min Yi, Min Yan, Yu Li, Hong-En Wang, Huan-Xin Gao, Li-Qiang Mai, Tawfique Hasan, Bai-Xiang Xu, Dong-Liang Peng, Gustaaf Van Tendeloo, Bao-Lian Su

**Affiliations:** State Key Laboratory of Advanced Technology for Materials Synthesis and Processing, Wuhan, University of Technology, Wuhan 430070, China; Cambridge Graphene Centre, University of Cambridge, Cambridge CB3 0FA, UK; State Key Laboratory of Advanced Technology for Materials Synthesis and Processing, Wuhan, University of Technology, Wuhan 430070, China; Nanostructure Research Centre, Wuhan University of Technology, Wuhan 430070, China; State Key Laboratory of Advanced Technology for Materials Synthesis and Processing, Wuhan, University of Technology, Wuhan 430070, China; Faculty of Materials Science and Chemistry, China University of Geosciences, Wuhan 430074, China; Institute of Materials Science, Technische Universität Darmstadt, Darmstadt 64287, Germany; State Key Laboratory of Advanced Technology for Materials Synthesis and Processing, Wuhan, University of Technology, Wuhan 430070, China; State Key Laboratory of Advanced Technology for Materials Synthesis and Processing, Wuhan, University of Technology, Wuhan 430070, China; State Key Laboratory of Advanced Technology for Materials Synthesis and Processing, Wuhan, University of Technology, Wuhan 430070, China; Fundamental Research Department, SINOPEC Shanghai Research Institute of Petrochemical Technology, Shanghai 201208, China; State Key Laboratory of Advanced Technology for Materials Synthesis and Processing, Wuhan, University of Technology, Wuhan 430070, China; Cambridge Graphene Centre, University of Cambridge, Cambridge CB3 0FA, UK; Institute of Materials Science, Technische Universität Darmstadt, Darmstadt 64287, Germany; Department of Materials Science and Engineering, College of Materials, Xiamen University, Xiamen 361005, China; Nanostructure Research Centre, Wuhan University of Technology, Wuhan 430070, China; Electron Microscopy for Materials Science, University of Antwerp, Antwerp B-2020, Belgium; State Key Laboratory of Advanced Technology for Materials Synthesis and Processing, Wuhan, University of Technology, Wuhan 430070, China; Laboratory of Inorganic Materials Chemistry, University of Namur, Namur B-5000, Belgium

**Keywords:** (001) faceted TiO_2_ nanosheets, reduced graphene oxide, porous hierarchy, unprecedented lithium storage capacity, Li_2_Ti_2_O_4_ crystallites

## Abstract

Active crystal facets can generate special properties for various applications. Herein, we report a (001) faceted nanosheet-constructed hierarchically porous TiO_2_/rGO hybrid architecture with unprecedented and highly stable lithium storage performance. Density functional theory calculations show that the (001) faceted TiO_2_ nanosheets enable enhanced reaction kinetics by reinforcing their contact with the electrolyte and shortening the path length of Li^+^ diffusion and insertion-extraction. The reduced graphene oxide (rGO) nanosheets in this TiO_2_/rGO hybrid largely improve charge transport, while the porous hierarchy at different length scales favors continuous electrolyte permeation and accommodates volume change. This hierarchically porous TiO_2_/rGO hybrid anode material demonstrates an excellent reversible capacity of 250 mAh g^–1^ at 1 C (1 C = 335 mA g^–1^) at a voltage window of 1.0–3.0 V. Even after 1000 cycles at 5 C and 500 cycles at 10 C, the anode retains exceptional and stable capacities of 176 and 160 mAh g^–1^, respectively. Moreover, the formed Li_2_Ti_2_O_4_ nanodots facilitate reversed Li^+^ insertion-extraction during the cycling process. The above results indicate the best performance of TiO_2_-based materials as anodes for lithium-ion batteries reported in the literature.

## Teaser

A hierarchically porous TiO_2_/rGO hybrid architecture with active (001) facets exhibits unprecedented and highly stable lithium storage performance for TiO_2_-based anodes.

## INTRODUCTION

Design of materials with high capacity, excellent rate capability and long cycle life is a major challenge in the field of rechargeable lithium-ion batteries (LIBs) [1–3]. Among the various anode materials, TiO_2_ is very promising because of its high activity, high abundance, nontoxicity and electrochemical and structural stability [4]. Importantly, TiO_2_ anodes offer safer operation as they avoid formation of solid electrolyte interphase layers in the voltage window 1.0–3.0 V [5]. However, the kinetics of Li^+^ insertion-extraction and the overall electrochemical performance of TiO_2_ anode materials are often limited by low Li^+^ diffusion and charge transport, as well as a low electrode/electrolyte contact area [6].

Various efforts have been made to enhance the reaction kinetics of Li^+^ diffusion and insertion-extraction [7–13]. Assembling low-dimensional nanostructures to construct hierarchical micro/nanostructures is a widely adopted strategy to shorten the pathway for Li^+^ diffusion and electron transport, and to increase the electrode/electrolyte contact area [14–19]. In particular, because of their high anisotropy and nanoscale thickness, two-dimensional (2D) anatase TiO_2_ nanosheets with (001) facets demonstrate a high capacity and excellent rate performance [8]. In addition, 2D TiO_2_ nanosheets with a high specific surface area can increase the active sites for Li^+^ insertion, offering pseudo-capacitive Li^+^ storage capability [18,19]. It has been reported recently that nanosheet-constructed yolk-shell TiO_2_ porous microspheres enable easy permeation and storage of electrolyte, and facilitate charge diffusion and Li^+^ insertion with outstanding endurance of the volume change during the Li^+^ insertion-extraction process; resulting in excellent reversible capacity, long cycle performance (>700 cycles) and superior rate capability [18]. The 2D (001) faceted TiO_2_ nanosheets are therefore quite promising as anode materials for design of supercapacitor-like LIBs with high energy and power densities [19,20]. However, the high energy of the (001) facets results in tight aggregation of the TiO_2_ nanosheets when fabricating the anodes. This reduces the access of Li^+^ to the active (001) facets and impedes electrolyte penetration inside the structure, deteriorating the electrochemical performances.

Here, we report the unprecedented lithium storage and electrochemical performance of a nanosheet-constructed hierarchically porous TiO_2_/rGO (NSTiO_2_/rGO) hybrid architecture. In the synthesis, TiF_4_ is used to ensure the formation and exposure of (001) faceted TiO_2_ nanosheets [21]. The flexible graphene oxide (GO) nanosheets, before their reduction, regulate growth and assembly of the (001) faceted TiO_2_ nanosheets. Meanwhile, GO is gradually converted into reduced graphene oxide (rGO) via isopropyl alcohol reduction during the TiO_2_ growth and assembly process [22]. The resulting hierarchically porous NSTiO_2_/rGO hybrid anode material offers a high and stable specific surface area (304.5 m^2^ g^–1^) and exhibits an excellent reversible capacity of 250 mAh g^–1^ at 1 C (1 C = 335 mA g^–1^), twice that of pure NSTiO_2_ without rGO. After 1000 cycles at 5 C, the reversible capacity is still stabilized at 176 mAh g^–1^. Even if the current density is increased to 10 C (∼5 minutes to a full capacity), a very stable and extraordinarily high reversible capacity of 160 mAh g^–1^ is achieved after 500 cycles. This result is the best performance reported so far with use of TiO_2_-based anodes for LIBs. The present work paves the way for pursuing optimized properties of active faceted hybrid micro/nanostructures and provides a very promising anode material for industrial application in high-performance LIBs.

## RESULTS AND DISCUSSION

**Figure 1.**
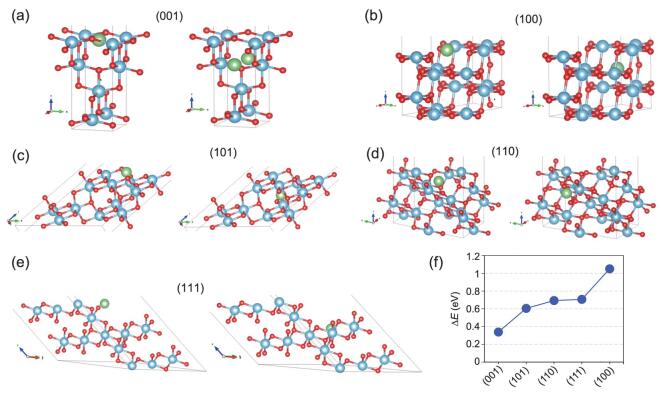
Crystal models for Li ions migrating from various crystal surfaces to the internal octahedral voids. Left (initial state) and right (final state): (a) (001), (b) (100), (c) (101), (d) (110) and (e) (111) crystal plane surfaces. (f) The calculated energy barriers for the corresponding crystal planes. Li ions are in green, Titanium atoms are in blue and oxygen atoms are in red.

Density functional theory calculations are first carried out to study the energy barriers for Li^+^ migration on the (001), (101), (110), (111) and (100) crystal planes of anatase TiO_2_. Figure 1a–e displays the crystal models of the different crystal surfaces. The energy barriers are calculated by the energy difference between the initial state (Li ions located on the surface by relaxation) and the final state (Li ions located in internal octahedral voids close to the surface, but with minimum energy). The calculated energy barriers are presented in Fig. 1f. The energy barrier of Li^+^ entering into the (001) surface of anatase TiO_2_ is the lowest, suggesting much easier migration of Li^+^ across the (001) surface.

**Figure 2.**
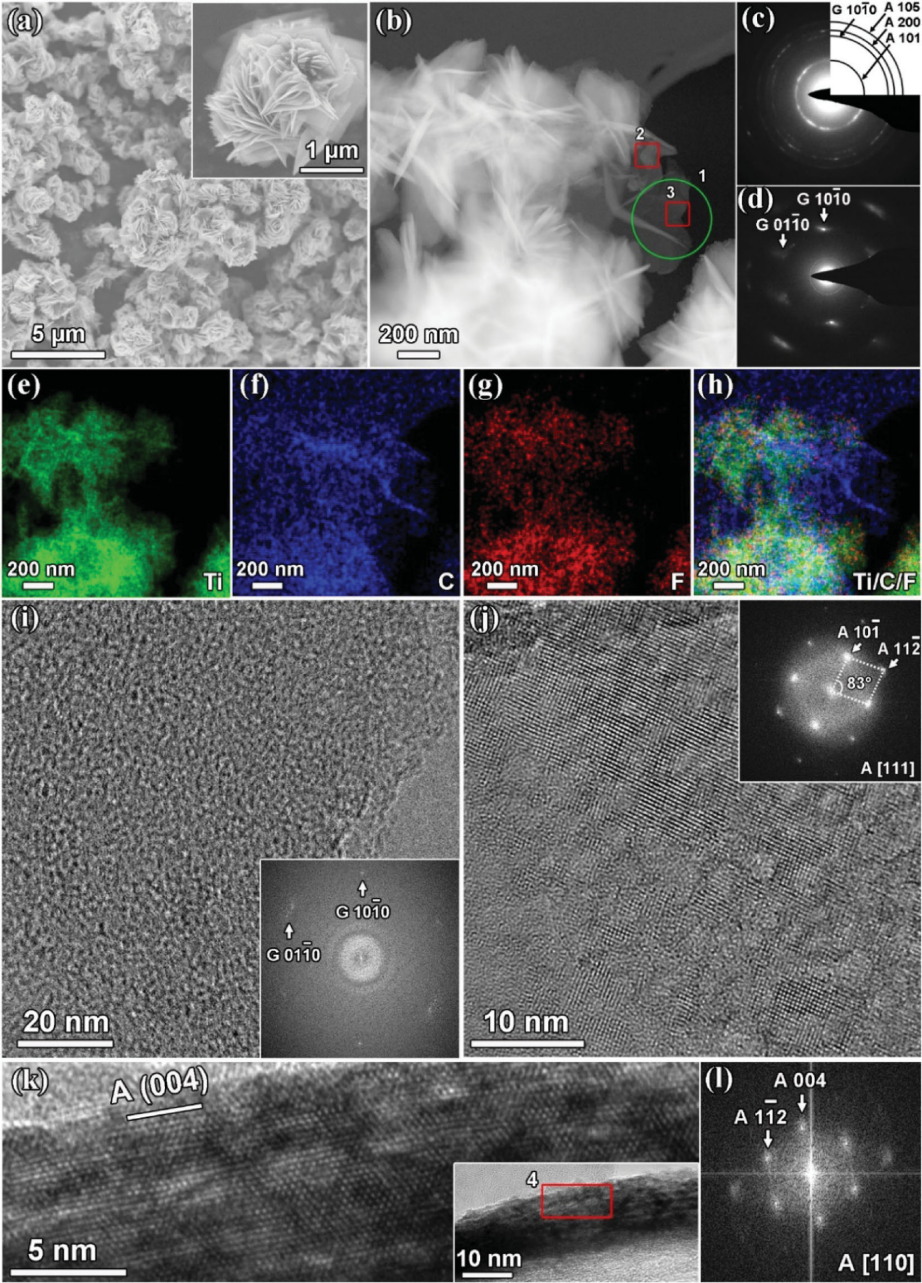
Electron microscopy characterization of the NSTiO_2_/rGO hybrid structure. (a) SEM images. (b) High angle annular dark field scanning transmission electron microscopy image. (c) SAED pattern of the whole area in (b). (d) SAED pattern of zone 1. (e–h) EDX maps of the whole area in (b). (i) HRTEM image of rGO in zone 3 and the corresponding FFT image (inset). (j) HRTEM image of anatase in zone 2 and the corresponding FFT image (inset). (k) Transmission electron microscopy (TEM) image of an anatase nanosheet (inset) and HRTEM image of zone 4. (l) The corresponding FFT of zone 4.

The synthesis and self-assembly processes of the NSTiO_2_ and the NSTiO_2_/rGO hybrid microflower structure are illustrated in Fig. S1. Without GO acting as a 2D scaffold directing the growth and self-assembly of NSTiO_2_, the (001) faceted TiO_2_ nanosheets are tightly aggregated and form ∼5 μm microspheres to reduce the surface energy (Figs S1b, S2–S4). After GO is added to the reaction system, TiF_4_ is first anchored onto the surface of the highly dispersed GO nanosheets via chemisorption on the oxygen-containing functional groups (e.g. –OH and –COOH) [22]. During the solvothermal growth, the TiO_2_ nanocrystallites gradually deposit onto the GO while F^–^ adsorbs on the TiO_2_ nanocrystallites to direct formation of the (001) faceted TiO_2_ nanosheets (Fig. S1a). The TiO_2_ nanosheets tend to aggregate to reduce their surface energy while the GO sheets prevent such aggregation. The TiO_2_ and GO nanosheets self-assemble into hierarchically porous hybrid microflowers (Fig. 2). During this process, isopropyl alcohol reduces the GO nanosheets to electrically conductive rGO nanosheets.

The crystalline structures of GO and NSTiO_2_/rGO were examined by X-ray diffraction (XRD) (Fig. S5). For the as-prepared GO and rGO, the peaks at 2θ = 12.1° and 2θ = 24.5° correspond to the (002) reflection of the stacked GO sheets and the (002) reflection of rGO, respectively. The diffraction pattern of NSTiO_2_/rGO exhibits distinctive peaks of anatase TiO_2_ (JCPDS No. 089–4921). No diffraction peaks of rGO are observed in this composite. This should be ascribed to its low content and weak diffraction intensity. It is also worth mentioning that the peak of rGO at 24.5° may be shielded by the peak of TiO_2_ at 25.3° [23]. In addition, strong and broader peaks compared to those of pure NSTiO_2_ are observed. The average grain sizes calculated from the Scherrer equation are 7.5 nm for NSTiO_2_/rGO and 18.6 nm for NSTiO_2_, respectively, indicating smaller and/or thinner TiO_2_ nanosheets in NSTiO_2_/rGO.

The scanning electron microscopy (SEM) image of NSTiO_2_/rGO in Fig. 2a shows that all NSTiO_2_/rGO hybrids have a flower-like morphology of size 1–2 μm, significantly smaller than that of pure NSTiO_2_ at a size of ∼5 μm. The magnified SEM image (Fig. 2a inset) confirms that the NSTiO_2_/rGO microflowers are constructed by the assembly of TiO_2_ nanosheets and rGO. Note that the transparent films in the inset of Fig. 2a are rGO nanosheets.

The hierarchical NSTiO_2_/rGO micro/nanostructure is revealed by high angle annular dark field scanning transmission electron microscopy (Fig. 2b). The corresponding selected area electron diffraction (SAED) of the whole area reveals the typical anatase TiO_2_ and graphene diffraction rings (Fig. 2c). The SAED of the edge zone (zone 1) shows a pure graphene diffraction pattern (Fig. 2d), suggesting that the microflowers grow on a support of rGO nanosheets. To further confirm the hybrid structure of TiO_2_ and rGO, the distribution of TiO_2_ and rGO is demonstrated by energy dispersive X-ray spectroscopy (EDX) mapping (Fig. 2e–h) over the same area of Fig. 2b. The titanium (Fig. 2e) and fluorine (Fig. 2g) maps confirm that the F^–^ is homogeneously distributed over the (001) facets of the TiO_2_ nanosheets in the hybrid microflowers (Fig. 2h).

To clarify the surface chemical states of NSTiO_2_/rGO composite, X-ray photoelectron spectroscopy was performed as exhibited in Fig. S6. The typical X-ray photoelectron spectroscopy spectrum of NSTiO_2_/rGO in Fig. S6a demonstrates the existence of C, Ti, O and F, consistent with the EDX mapping results. Fig. S6b and S6c indicate that no carbon doping reaction and Ti^3+^ defect occur during the hydrothermal process. In Fig. S6d, the F 1s peak at 684.3 eV is attributed to ≅Ti-F configuration and no signal of fluorine substituting for surface bridging oxygen (688.5 eV) is detected [24,25]. Such a bond can reduce the charge transfer resistance of electrodes because of its high electronegativity and the surface F can rapidly produce a large number of LiF during the discharge process [26]. The grain boundaries among LiF facilitate uniform diffusion of Li^+^ through the solid–electrolyte interphase and contribute to stable interphase generation [27]. However, the amount of F in the NSTiO_2_/rGO is only 5.9 at%, much lower than that of the precursor solution, and its contribution to lithium storage capacity is still limited. Confirming the SAED from zone 1 (Fig. 2d), the distribution of rGO can be distinguished in the carbon map (Fig. 2f). This indicates that the TiO_2_ nanosheets are overlaid onto the rGO nanosheets, consistent with the SEM observation. The HRTEM (Fig. 2i) and the corresponding FFT (Fig. 2i inset) in the edge zone 3 of the NSTiO_2_/rGO structure present a weak graphene hexagonal structure because of partial reduction. The HRTEM and FFT of Fig. 2j in the center zone 2 (Fig. 2b) demonstrate an intermediate state of crystal growth from TiO_2_ nanocrystallites to nanosheets. This suggests that the TiO_2_ nanocrystallites first nucleate and then transform to (001) faceted nanosheets via the regulation of F^–^ and rGO nanosheets [22,28]. Figure 2k–l show one TiO_2_ nanosheet with a thickness of 10 nm and confirm that the NSTiO_2_/rGO structure has a highly exposed (001) facet.

Raman spectroscopy is used to confirm the sp^2^ and sp^3^ hybridization of carbon atoms in NSTiO_2_/rGO. The symmetry-allowed E_2g_ mode of sp^2^-bonded carbon atom at the Γ-point, commonly termed the G-band, is observed at ∼1588 cm^–1^ (Fig. S7a). The D-band at ∼1328 cm^–1^ is related to the vibration of sp^3^-hybridized carbon atoms near the K-point and is usually associated with disorder or defects (such as those arising from oxidation) in the graphene lattice [29]. The high I_D_/I_G_ ratio indicates an increase of defects in the samples [29]. The other three peaks at 393, 510 and 625 cm^–1^ are characteristic of the B_1g_, A_1g_ and E_g_ modes of anatase TiO_2_ [30,31]. The content of rGO in NSTiO_2_/rGO is measured to be 10.81% by thermogravimetric analysis (Fig. S7b).

**Figure 3.**
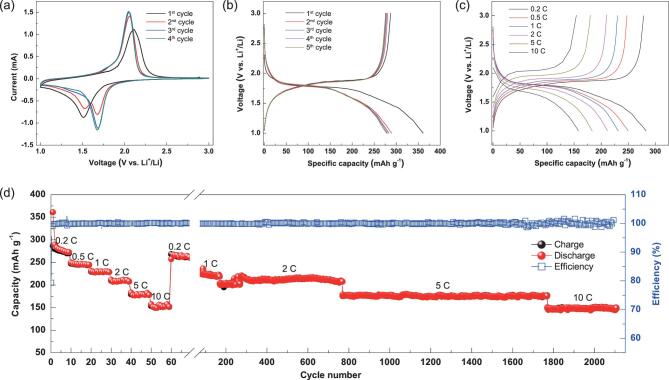
Electrochemical characterization of the NSTiO_2_/rGO anode. (a) Representative CV curves at a scan rate of 0.2 mV s^–1^. (b) Discharge-charge profiles at 0.2 C. (c) Discharge-charge profiles at various current densities. (d) The rating and cycling performances at various current densities.

The specific surface area and pore size distribution of NSTiO_2_/rGO are characterized by N_2_ adsorption-desorption (Fig. S7c and d). The adsorption-desorption curve exhibits a type-II isotherm with a pore size distribution centered at 13 nm. The hysteresis loop at high p/p_o_ from 0.5 to 1.0 indicates the presence of macropores, suggesting the existence of meso-macroporous hierarchy in the material. NSTiO_2_/rGO exhibits a high specific surface area of 304.5 m^2^ g^–1^ and an adsorption cumulative volume of 1.58 cm^3^ g^–1^. In comparison, NSTiO_2_ exhibits a very low specific surface area of 13.5 m^2^ g^–1^. The high surface area and mesoporous structure in the hierarchically porous NSTiO_2_/rGO micro/nanostructure

could be very beneficial for Li^+^ storage. Figure 3a displays cyclic voltammograms (CVs) of the NSTiO_2_/rGO electrode at 0.2 mV s^–1^. In the first cycle, two well-defined peaks are observed at ∼1.51 (cathodic sweep) and ∼2.11 V (anodic sweep). In the second cycle, the intensity of the cathodic peak at ∼1.51 V decreases while a new cathodic peak with higher intensity appears at ∼1.68 V. Also, the anodic peak shifts to ∼2.06 V with increased intensity. In the third cycle, the cathodic peak at ∼1.51 V disappears and the peak intensity at ∼1.68 V enhances significantly. The enhanced peak profiles of NSTiO_2_/rGO are narrower, with a high peak current after three cycles. This indicates decreased polarization of the NSTiO_2_/rGO electrode, revealing an easy electrochemical reversible reaction of Ti^3+^ to Ti^4+^ during the Li^+^ insertion-extraction process because of the higher electrical conductivity of rGO and, furthermore, enhanced Li^+^ insertion-extraction kinetics in NSTiO_2_/rGO. After the second cycle, the CV sweep curves remain unchanged, indicating an excellent reversible stability of the NSTiO_2_/rGO electrode. This is quite different from the CVs of NSTiO_2_, in which the anodic and cathodic peaks always change for the first four cycles (Fig. S8a), indicating unstable capability for the LIBs.

Figure 3b depicts the discharge-charge profiles of the NSTiO_2_/rGO electrode for the first five cycles at 0.2 C (1 C = 335 mA g^–1^). Two well-defined voltage plateaus appear at ∼1.8 and ∼1.9 V during the discharge and charge processes, consistent with the CV analysis. The NSTiO_2_/rGO demonstrates an initial discharge capacity of 360 mAh g^–1^ and a subsequent charge capacity of 290 mAh g^–1^, giving a high initial coulombic efficiency of 80.6%. The discharge and charge curves can be divided into three stages. For the first stage of the discharge process in the first cycle, the potential drops from the open circuit value of ∼2.3 V to a value of ∼1.85 V with a Li^+^ insertion capability of 34 mAh g^–1^. The second stage is the horizontal plateau region, which reflects the process of Li^+^ insertion into the vacant sites of the TiO_2_ crystal structure, with a Li^+^ insertion capability of 180 mAh g^–1^. The last stage is the gradual decay of the voltage after the plateau region. This reflects the insertion process of Li^+^ into the surface layer of the anode material, with a Li^+^ insertion capability of 147 mAh g^–1^. The discharge and charge capacities in the second cycle are 289 and 281 mAh g^–1^, respectively, with a high coulombic efficiency of 97.2%. The coulombic efficiencies in the third, fourth and fifth cycles are 98.2%, 98.7% and 99.0%, respectively. The continuously increased coulombic efficiency indicates the reversible stability and fast balance of the Li^+^ insertion-extraction, resulting from the (001) faceted nanosheet-constructed hierarchical micro/nanostructure and rGO layers [8,32]. The NSTiO_2_ demonstrates a lower initial discharge capacity of 223 mAh g^–1^ and a subsequent charge capacity of 180 mAh g^–1^ (Fig. S8b), and its initial coulombic efficiency reaches as high as 80.7%. The capacities of the subsequent cycles are very stable with high coulombic efficiencies, confirming that the (001) facets are beneficial for Li^+^ insertion-extraction. Compared to pure NSTiO_2_, the NSTiO_2_/rGO with a much higher surface area intensively increases the active sites for Li^+^ insertion-extraction, its porous structure will significantly facilitate electrolyte permeation and largely endurance volume expansion; its thinner nanosheet constructed porous network could notably shorten the path length for Li^+^ insertion-extraction and the rGO nanosheets increase the charge transfer, leading to a highly enhanced capacity.

Figure 3c displays the discharge-charge profiles of the NSTiO_2_/rGO electrode at various rates. The discharge capacities are 283, 250, 232, 212, 183 and 160 mAh g^–1^ at 0.2, 0.5, 1, 2, 5 and 10 C, respectively. When the current density is 0.2 C, the capacities of the first, second and third stages are 34, 119 and 137 mAh g^–1^, respectively. The capacities of the first, second and third stages are 35, 111 and 105 mAh g^–1^ at 0.5 C; 35, 107 and 89 mAh g^–1^ at 1 C; 34, 85 and 93 mAh g^–1^ at 2 C; 36, 57, 91 mAh g^–1^ at 5 C, respectively (Table S1). From the above data, the decreased discharge capacities from 0.2 to 1 C primarily come from the capacity changes at the third stage. On the other hand, the decreased discharge capacities from 1 C to 5 C are mainly affected by the capacity changes at the second stage. The higher kinetic efficiency of Li^+^ insertion to the additional surface (at the third stage) rather than that to the crystal structure (at the second stage) at high current rates is the primary reason for this phenomenon. At low current densities (<1 C), the (001) facets of TiO_2_ facilitate and dominate the Li^+^ insertion-extraction, whereas at high current densities (>1 C), the high specific surface area and porosity in the hierarchically porous NSTiO_2_/rGO architecture dominate the Li^+^ insertion-extraction. Without rGO (i.e. for NSTiO_2_), capacities at both the second and third stages decrease very fast under increased current density (Fig. S8c), because the low specific surface area and tight aggregation of (001) faceted TiO_2_ nanosheets impede electrolyte penetration inside the structure.

Figure 3d shows the rate and cycle performance of the NSTiO_2_/rGO electrode at various rates. When discharged at 0.2 C, the NSTiO_2_/rGO has an initial discharge capacity of 360 mAh g^–1^ and a subsequent charge capacity of 290 mAh g^–1^. After 10 cycles, the NSTiO_2_/rGO anode exhibits a discharge capacity of 273 mAh g^–1^ and a subsequent charge capacity of 271 mAh g^–1^, leading to a very high coulombic efficiency of 99.3%. When the current densities are increased to 0.5, 1, 2, 5 and 10 C, the discharge capacities are decreased to 250, 232, 212, 183 and 160 mAh g^–1^, respectively. As the current rate is set back to 0.2 C, the discharge capacity of the NSTiO_2_/rGO is again increased to 268 mAh g^–1^. Following this, with the same unit cell, a reversible charge capacity of 225 mAh g^–1^ is retained after 100 cycles at 1 C. A reversible charge capacity of 212 mAh g^–1^ is achieved after 500 cycles at 2 C. When the current density is further increased to 5 C, the reversible charge capacity achieved is 176 mAh g^–1^ after 1000 cycles. Our NSTiO_2_/rGO anode material demonstrates the best performance compared to all the TiO_2_ and graphene-TiO_2_ anode materials reported to date (Table 1) [33–44]. In addition, the coulombic efficiency remains at ∼100%. Even at 10 C for more than 500 cycles, the reversible charge capacity stabilizes at 160 mAh g^–1^. Such ultrahigh electrochemical performance and ultralong cycle life can be attributed to the hierarchically porous (001) faceted nanosheet-constructed micro/nanostructure with flexible and conductive rGO nanosheets. Without rGO addition, NSTiO_2_ demonstrates very low capacities at different rates because of the tight aggregation of (001) faceted TiO_2_ nanosheets (Fig. S8d).

**Table 1.** Comparison of the electrochemical performance of NSTiO_2_/rGO with other reported high-performance TiO_2_ materials.

	Capacity (mAh g^–1^) at different rates (mA g^–1^)						
Electrode materials	100	200	500	1000	2000	Cycling performance (mAh g^–1^)	Ref.
TiO_2_-NCF	203	188	169	147	104	149, 100 cycles at 1 A g^–1^	[33]
TiO_2_-GAs	202	165	150	135	110	200, 50 cycles at 0.59 C	[34]
TiO_2_-G	–	162/1 C	–	130/5 C	123/10 C	180, 30 cycles at 0.2 C	[35]
TiO_2_-Cu	180/0.5 C	145/1 C	–	70/5 C	50/10 C	115, 100 cycles at 1 C	[36]
TiO_2_-NS	215	197	–	–	–	140, 200 cycles at 400 mA g^–1^	[37]
TiO_2_-GNS	170	145	115	100	75	60, 400 cycles at 5 A g^–1^	[38]
TiO_2_-N-rGO	220/0.5 C	175/1 C	150/2 C	–	125/10 C	127, 100 cycles at 10 C	[39]
TiO_2_-H	–	185/1 C	150/2 C	115/5 C	85/10 C	187, 300 cycles at 1 C	[40]
TiO_2_-rGO (10%)	208/0.5 C	186/1 C	164/2 C	145/5 C	127/10 C	174, 200 cycles at 1 C	[41]
C@TiO_2_@C	193/0.5 C	166/1 C	146/2 C	115/5 C	–	191, 200 cycles at 0.2 C	[42]
TiO_2_-rGO	200/0.5 C	180/1 C	160/2 C	150/5 C	140/10 C	157, 1000 cycles at 10 C	[43]
TiO_2_/rGO	303/0.5 C	229/1 C	200/2 C	185/5 C	171/10 C	131, 1000 cycles at 10 C	[44]
NSTiO_2_/rGO	250/0.5 C	232/1 C	212/2 C	183/5 C	160/10 C	160, 500 cycles at 10 C	This work^a^

^a^1 C = 335 mA g^–1^ for this work, 1 C = 168 mA g^–1^ for other work.

As a comparison, the cycling performance at 1 C and rating performance at various current densities of rGO are displayed in Fig. S7e and f. Considering the low content of rGO, its contribution percentage of the capacity of NSTiO_2_/rGO at 0.2 C (∼290 mAh g^−1^) is only 3.0%. When the current density is increased to 1 C, the reversible charge capacity of rGO is ∼50 mAh g^−1^ and its contribution percentage of NSTiO_2_/rGO (∼232 mAh g^−1^) decreases to 2.3%. This indicates that the addition of flexible rGO nanosheets is very important to regulate the formation of (001) faceted nanosheet-constructed porous hybrid microflowers and to enhance the electrochemical performance of anatase TiO_2_. The mediation role of TiF_4_ for the formation of (001) faceted TiO_2_ nanosheets and the insertion of GO between nanosheets is essential for the fabrication of such unprecedented high-performance material.

**Figure 4.**
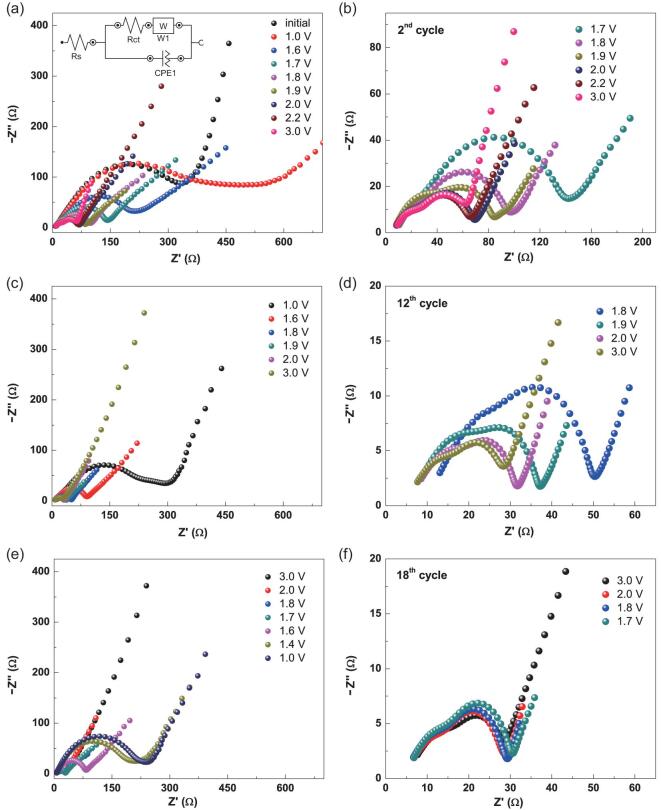
EIS spectra of NSTiO_2_/rGO anode material during the charge-discharge processes for different cycles (1.0–3.0 V). (a, b) EIS spectra of the fresh electrode and the electrode during the charge process at the second cycle. (c, d) EIS spectra of the electrode during the charge process at the 12th cycle. (e, f) EIS spectra of the electrode during the discharge process at the 18th cycle. The inset in (a) is the simulated circuit.

Electrochemical impedance spectroscopy (EIS) is used to investigate Li^+^ insertion-extraction in the NSTiO_2_/rGO anode material. Figure 4 shows the Nyquist plots of the initial electrode and the same electrode after the discharge-charge process at different cycles. The spectra are analyzed and fit with an equivalent circuit model in the inset of Fig. 4a. The high-medium frequency regions of the semicircles are ascribed to the surface layer resistance R_s_ (the first semicircle) at the surface layer and the charge-transfer resistance R_ct_ (the second semicircle) in the electrode/electrolyte interface, while the low-frequency region of the straight line corresponds to the diffusion of Li^+^ into the anode material (Warburg diffusion) [19]. The fresh NSTiO_2_/rGO anode shows a surface resistance R_s_ = 14.8 Ω and a charge-transfer resistance R_ct_ = 386 Ω, respectively (Fig. 4a and b and Table S2). With a potential of 1.0 V at the full discharge-state (the second cycle), the NSTiO_2_/rGO electrode demonstrates R_s_ = 15.1 Ω and R_ct_ = 477.9 Ω, respectively. During the charging process, R_s_ and R_ct_ values gradually decrease as the potential increases to 3.0 V, eventually reaching 6.1 Ω and 74.2 Ω at a potential of 3.0 V. After 12 cycles, the NSTiO_2_/rGO electrode exhibits R_s_ = 16.6 Ω and R_ct_ = 241 Ω at a potential of 1.0 V (Fig. 4c and d and Table S3). Thus, the surface layer resistance R_s_ remains virtually unchanged whereas the charge-transfer resistance R_ct_ decreases significantly. This indicates that the activation process of the NSTiO_2_/rGO anode is consistent with the CV analysis (Fig. 3a).

Figure 4e and f present the EIS spectra of the NSTiO_2_/rGO anode at the 18th cycle of the discharge process. The R_s_ and R_ct_ values of NSTiO_2_/rGO anode at the fully charged state are 5.3 and 23.5 Ω, respectively (Table S4). After full discharge, R_s_ and R_ct_ increase to 7.5 and 225 Ω. It is well known that Rs is determined by the space charge layer [45], thus the values of R_s_ can keep stable because of the limited lattice space for Li^+^ insertion/extraction and the charge density does not increase dramatically. R_ct_ of the battery is determined by the exchange current and is given by ${\rm R_{\rm ct}} = {^{\rm RT}} /_{\rm F \times {I_0}}$ (R is gas constant, F is Faraday constant and I_0_ is exchange current) and/or it can also be calculated by the equation ${\rm R_{ct}}\sim\frac{1}{{{{( {\rm SOC} )}^{\alpha} }{{( {\rm 1 - SOC} )}^{\alpha} }}}$ (SOC is State-of-Charge, $\alpha$ is transfer coefficient and $\alpha$ = 0.5 for this anode) [46]. During the discharge process of NSTiO_2_/rGO from 3.0 V to 1.0 V, the concentration of SOC decreases and the R_ct_ increases continuously. This indicates stable R_s_ and inverse R_ct_ during the discharge-charge cycling process from the NSTiO_2_/rGO porous hybrid structure. Figure S9 shows the EIS spectra of NSTiO_2_ and NSTiO_2_/rGO (charged to 3.0 V) after 100 cycles at 1 C. The R_s_ of NSTiO_2_ is almost unchanged compared to that of NSTiO_2_/rGO. It also clearly demonstrates that NSTiO_2_/rGO has a much lower R_ct_ (26.9 Ω) than NSTiO_2_ (62.2 Ω) (Table S5). Further, the slope of the straight line in the low-frequency region (Warburg diffusion) of NSTiO_2_/rGO is much higher than that of NSTiO_2_, indicating much faster Li^+^ diffusion in the NSTiO_2_/rGO anode material. The extraordinary performance of NSTiO_2_/rGO can be attributed to the (001) faceted nanosheet-constructed porous flower-like structure ensuring good contact with the electrolyte and the conductive rGO nanosheets, guaranteeing fast charge transfer inside the structure [35,47,48].

**Figure 5.**
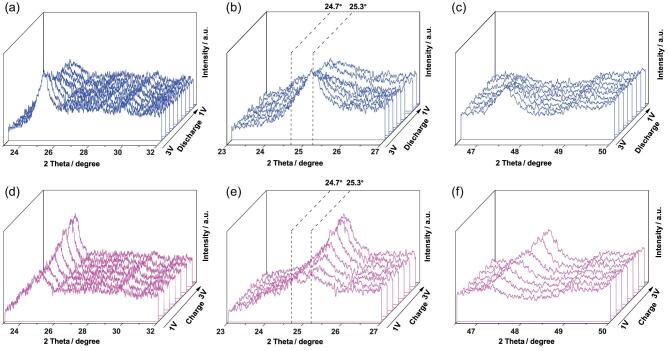
In situ XRD patterns of the NSTiO_2_/rGO during the discharge-charge processes. (a–c) Discharge process from 3.0 to 1.0 V. (d–f) Charge process from 1.0 to 3.0 V.

The in situ XRD measurements provide more insight into the observed electrochemical transitions of the NSTiO_2_/rGO anode material. The lower current density of 0.2 C is selected to trace details of the structure conversion during the discharge-charge processes. As shown in Fig. 5a, with the Li^+^ insertion into the anode material, the intensity of the Bragg peaks of the initial anatase phase gradually decreases. A more detailed observation on the intense reflection (101) at 25.3° reveals that a new peak at 24.7° appears when the voltage is ∼2.0 V (Fig. 5b). Meanwhile no obvious shift of the (200) reflection at 46.7° is observed (Fig. 5c). This phenomenon can be attributed to a solid solution domain followed by a biphasic transition [49,50]. Figure 5d presents the in situ XRD patterns during the charge process. The intensity of peaks at 25.3° and 46.7° increases and the peak at 24.5° declines until it completely disappears from 1 V to 3 V (Fig. 5e and f). The recovered peak at 25.3° is still narrow and intense, indicating that the (001) faceted NSTiO_2_/rGO microflowers have a great reversible extraction capacity during the discharge-charge process and endure fast transfer inside the anode material.

Post-mortem studies after 100 cycles at 1 C are carried out to further reveal the structural stability and lithium storage property of the anode material through SEM and TEM observations. For this, the NSTiO_2_/rGO anode is immersed in acetone for more than 1 week to wash off the electrolyte. Figure S10a–c shows

that the hierarchically porous NSTiO_2_/rGO micro/nanostructure is generally maintained after the electrochemical reaction, confirming the structural and electrochemical stability of the anode material. Some flexible rGO nanosheets can be found wrapping around the TiO_2_ nanosheets (Figs S11 and S12), ensuring the long cycle life and superior rate performance of NSTiO_2_/rGO. Many uniform nanoparticles (∼5 nm) are randomly distributed on the surface of NSTiO_2_/rGO, in particular on the TiO_2_ nanosheets (Fig. S10d). These nanoparticles are cubic Li_2_Ti_2_O_4_ nanocrystallites (space group: *F3m3*, lattice constants: a = b = c = 8.375 Å), according to the corresponding SAED and HRTEM images (Fig. S10e and f). Therefore, the reactions in the TiO_2_/Li half-cell can be written as follows:

(1) \begin{equation*}\rm Ti {O_2} + xL{i^ + } + x{e^ - } \leftrightarrow L{i_x}Ti {O_2},\end{equation*}

(2) \begin{eqnarray*}\rm L{i_x}Ti {O_2} &+& \rm 2(1 - x)L{i^ + }\nonumber\\ &+&\rm 2(1 - x){e^ - } \leftrightarrow L{i_2}T{i_2}{O_4}.\end{eqnarray*}

**Figure 6.**
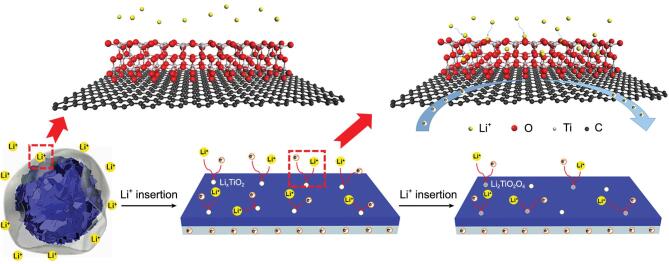
Schematic illustration of Li^+^ in (001) facet of NSTiO_2_/rGO.

Figure 6 illustrates that continuous Li^+^ insertion into the surface of the (001) faceted nanosheets will lead to an atomic rearrangement to form new cubic Li_2_Ti_2_O_4_ nanocrystals with one Li^+^ inserted per formula unit of TiO_2_. The Li_2_Ti_2_O_4_ islands further facilitate the Li^+^ insertion capability [51,52]. As the Li_2_Ti_2_O_4_ nanodots are found on the surface of TiO_2_ nanosheets, the formation of Li_2_Ti_2_O_4_ will mostly contribute to the additional surface capacity at the third stage. This further ensures the excellent capability, superior rate performance and long cycle life of the NSTiO_2_/rGO anode.

## CONCLUSION

We have developed an rGO assisted one-pot solvothermal alcoholysis process to engineer a hierarchical NSTiO_2_/rGO porous micro/nanostructured hybrid for advanced lithium storage. The (001) faceted TiO_2_ nanosheets are grown in situ on the rGO surface, and self-assemble into a hierarchical micro-nanostructure with good mechanical stability and high specific surface area. Our hybrid NSTiO_2_/rGO material demonstrates excellent capacity, long cycle life and superior rate capability through facilitation of continuous intercalation of Li^+^ into TiO_2_ and improved electron conductivity from the rGO. Li_2_Ti_2_O_4_ nanocrystals formed on the rGO further facilitate the surface capacity for high-performance LIBs. All these performances are much better than the state-of-the-art values reported in the literature using TiO_2_-based materials as anode materials. It is envisioned that this hierarchically porous NSTiO_2_/rGO micro/nanostructure hybrid can be used as an anode material for industrial application in high-performance LIBs and may be employed in other applications, such as supercapacitors, photocatalytic water splitting and solar cells, making our strategy a universal route towards design of active faceted hybrid micro/nanostructures.

## METHODS

### Synthesis of NSTiO_2_ microspheres

All reagents and solvents are of analytical grade and are used without any further purification. In a typical synthesis, 0.2 g TiF_4_ is added to 80 mL isopropyl alcohol. After stirring for 30 min, the resulting solution is transferred into a 100 mL Teflon-lined stainless steel autoclave. The temperature in the autoclave is maintained at 200°C for 24 h and then cooled to room temperature naturally. The obtained blue precipitate is filtered and washed with ethanol and distilled water several times. To study the effect of TiF_4_ in the growth process, various amounts of TiF_4_ are used.

### Synthesis of NSTiO_2_/rGO microspheres

GO sheets are prepared from natural graphite powder through a modified hummers method [53]. The GO powder is obtained through a freeze-drying process from the GO suspension. 15 mg GO powder is first dispersed in 80 mL isopropyl alcohol by ultrasonic treatment for 2 h. Then, 0.4 g TiF_4_ is introduced into the GO dispersion at room temperature. The mixture is ultrasonicated for 30 min and transferred to a 100 mL Teflon-lined stainless steel autoclave. The autoclave temperature is maintained at 200°C for 24 h, before being naturally cooled down to room temperature. The obtained dark blue precipitate is filtered and washed with ethanol and distilled water several times.

### Characterizations

XRD patterns are obtained using a Bruker D8 with Cu Κα radiation (λ = 0.15405 nm) at 40 mA and 40 kV. SEM is carried out using an S-4800 field emission SEM (FESEM, Hitachi, Japan). TEM, SAED, scanning transmission electron microscopy and EDX were performed on an FEI Tecnai Osiris electron microscope fitted with Super-X window-less EDX detector system, operated at 200 kV. Nitrogen adsorption-desorption isotherms are obtained using a Tri-Star surface area and porosity analyzer (Tri-Star II 3020) at 77 K. The specific surface area is calculated with the Brunauer-Emmett-Teller method. The pore size distribution is calculated with the Barret-Joyner-Halenda method. Thermogravimetric analysis and differential scanning calorimeter curves are recorded using a thermal analyzer (Setaram Labsys Evo) in the air with a temperature ramp rate of 5°C min^–1^ from room temperature. Raman measurements are carried out at room temperature, using an Invia Raman Microscope with 632.8 nm excitation source.

### Electrochemical characterization

Electrochemical experiments are performed with coin-type cells with pure lithium as both the counter electrode and the reference electrode at room temperature. The working electrode consists of the active material, the conductive agent (carbon black, super-P) and the polymer binder (poly(vinylidene difluoride)) in an 8:1:1 weight ratio. After these materials are thoroughly mixed in N-methyl-2-pyrrolidone, the as-prepared slurry is coated onto a Cu foil, before being dried at 120°C in a vacuum oven for 12 h. A circular disk electrode is punched from the foil and used as the working electrode. The electrolyte used is 1.0 M LiPF_6_ in a 50:50 (w/w) mixture of ethylene carbonate and diethyl carbonate. The mass loading of the NSTiO_2_/rGO electrode is 1.4–1.6 mg cm^−2^ and the thickness is ∼50 μm in a cell as shown in Fig. S13. The cell assembly is carried out in an Ar-filled glove box. Cyclic voltammetry (1–3 V) is performed using an electrochemical workstation (CHI 660D) at a scanning rate of 0.2 mV s^–1^.  The discharge-charge tests are performed using a multichannel battery testing system (LAND CT2001A) with a voltage window of 1–3 V at various densities. EIS is measured with an electrochemical workstation (Autolab PGSTAT 302 N) in a frequency range of 100 kHz to 10 mHz.

### Computational calculations

The energy barriers for Li ions migrating from the (001), (101), (110), (111) and (100) surfaces to the internal octahedral voids were estimated in the slab model through density functional theory calculations using VASP (Vienna Ab initio Simulation Package). The generalized gradient approximation was employed with the exchange-correlation functional of Perdew-Burke-Ernzerhof. The cutoff energy was set to be 500 eV. For relaxation of the slab model, the convergence criteria for the maximum force on each atom and the total energy were 0.02 eV/Å and 5 × 10^−6 ^eV, respectively. In the self-consistent calculations of the total energy, an energy convergence criterion of 10^−6 ^eV was used. The energy barriers were calculated by the energy difference between the initial state (Li ions located on the surface by relaxation) and the final state (Li ions located in internal octahedral voids close to the surface, but with the minimum energy): ΔE = E^final^ - E^initial^.

## Supplementary Material

nwaa028_Supplemental_File
