# Supplementary material for: Unprecedented and highly stable lithium storage capacity of (001) faceted nanosheet-constructed hierarchically porous TiO2/rGO hybrid architecture for high-performance Li-ion batteries
Source: Natl Sci Rev. 2020 Feb 20;7(6):1046–58. doi: 10.1093/nsr/nwaa028 (PMC8288978; doi:10.1093/nsr/nwaa028)
Supplement: nwaa028_Supplemental_File [file nwaa028_supplemental_file.docx]

Supporting Information

**Unprecedented and highly stable lithium storage capacity of (001) faceted nanosheet-constructed hierarchically porous TiO_2_/rGO hybrid architecture for high performance Li ions battery**

Wen-Bei Yu,^1,2†^ Zhi-Yi Hu,^1,3†^ Jun Jin,^1,4*^ Min Yan,^1^ Min Yi,^5^ Yu Li,^1*^ Hong-En Wang,^1^ Huan-Xin Gao,^6^ Tawfique Hasan,^2^ Bai-Xiang Xu,^5^ Dong-Liang Peng,^7^ Gustaaf Van Tendeloo^3,8^, Li-Qiang Mai^1^ and Bao-Lian Su^1,9*^

*^1^State Key Laboratory of Advanced Technology for Materials Synthesis and Processing, Wuhan University of Technology, 122 Luoshi Road, Wuhan 430070, China; Email:* [*yu.li@whut.edu.cn*](mailto:yu.li@whut.edu.cn)*;* [*baoliansu@whut.edu.cn*](mailto:baoliansu@whut.edu.cn)

*^2^ Cambridge Graphene Centre, University of Cambridge, 9 JJ Thomson Avenue, Cambridge CB3 0FA, United Kingdom*

*^3^ Nanostructure Research Centre (NRC), Wuhan University of Technology, 122 Luoshi Road, 430070, China.*

*^4^ Faculty of Materials Science and Chemistry, China University of Geosciences, Wuhan, 430074, China; Email:*

[*jinjun@cug.edu.cn*](mailto:jinjun@cug.edu.cn)

*^5^ Institute of Materials Science, Technische Universität Darmstadt, Darmstadt, 64287, Germany.*

*^6^ Fundamental Research Department, SINOPEC Shanghai Research Institute of Petrochemical Technology, 165 Pudong Bei Road, Shanghai 201208, China.*

*^7^ Department of Materials Science and Engineering, College of Materials, Xiamen University, 422 Siming South Road, Xiamen 361005, China.*

*^8^ EMAT (Electron Microscopy for Materials Science), University of Antwerp, 171 Groenenborgerlaan, B-2020 Antwerp, Belgium.*

*^9^ Laboratory of Inorganic Materials Chemistry (CMI), University of Namur, 61 rue de Bruxelles, B-5000 Namur, Belgium; E-mail:* [*bao-lian.su@unamur.be*](mailto:bao-lian.su@unamur.be)

† These authors contributed equally to this work.

**Table S1.** The capacities of NSTiO_2_/rGO at the total, 1^st^, 2^nd^ and 3^rd^ stages.

| **Current density** | **0.2 C** | **0.5 C** | **1 C** | **2 C** | **5 C** |
| --- | --- | --- | --- | --- | --- |
| **Total capacity (mAhg^–1^)** | 283 | 250 | 232 | 212 | 183 |
| **1st stage (mAhg^–1^)** | 34 | 35 | 35 | 34 | 36 |
| **2nd stage (mAhg^–1^)** | 119 | 111 | 107 | 85 | 57 |
| **3rd stage (mAhg^–1^)** | 137 | 105 | 89 | 93 | 91 |

**Table S2.** The values of R_s_ and R_ct_ in the EIS spectra of NSTiO_2_/rGO at different states at the 2^nd^ cycle.

| **Curve** | **R_s_ (Ω)** | **R_ct_ (Ω)** |
| --- | --- | --- |
| **initial** | 14.8 | 386.0 |
| **1.0 V** | 15.1 | 477.9 |
| **1.6 V** | 14.5 | 218.0 |
| **1.7 V** | 12.3 | 139.0 |
| **1.8 V** | 8.3 | 95.5 |
| **1.9 V** | 5.5 | 84.2 |
| **2.0 V** | 7.1 | 66.2 |
| **2.2 V** | 6.7 | 65.6 |
| **3.0 V** | 6.1 | 74.2 |

**Table S3.** The values of R_s_ and R_ct_ in the EIS spectra of NSTiO_2_/rGO at different states at the 12^th^ cycle.

| **Curve** | **R_s_ (Ω)** | **R_ct_ (Ω)** |
| --- | --- | --- |
| **1.0 V** | 16.6 | 24.1 |
| **1.6 V** | 9.4 | 85.3 |
| **1.8 V** | 11.7 | 40.8 |
| **1.9 V** | 7.5 | 31.2 |
| **2.0 V** | 7.1 | 26.1 |
| **3.0 V** | 5.3 | 23.5 |

**Table S4.** The values of R_s_ and R_ct_ in the EIS spectra of NSTiO_2_/rGO at different states at the 18^th^ cycle.

| **Curve** | **R_s_ (Ω)** | **R_ct_ (Ω)** |
| --- | --- | --- |
| **3.0 V** | 5.3 | 23.5 |
| **2.0 V** | 6.0 | 24.5 |
| **1.8 V** | 5.9 | 24.6 |
| **1.7 V** | 6.0 | 26.6 |
| **1.6 V** | 8.9 | 76.7 |
| **1.4 V** | 5.3 | 206.0 |
| **1.0 V** | 7.5 | 225.0 |

**Table S5.** The values of R_s_ and R_ct_ at the full charge state (3.0 V) of NSTiO_2_ and NSTiO_2_/rGO.

| **Sample** | **R_s_ (Ω)** | **R_ct_ (Ω)** |
| --- | --- | --- |
| **NSTiO_2_** | 5.1 | 62.2 |
| **NSTiO_2_/rGO** | 5.4 | 26.9 |


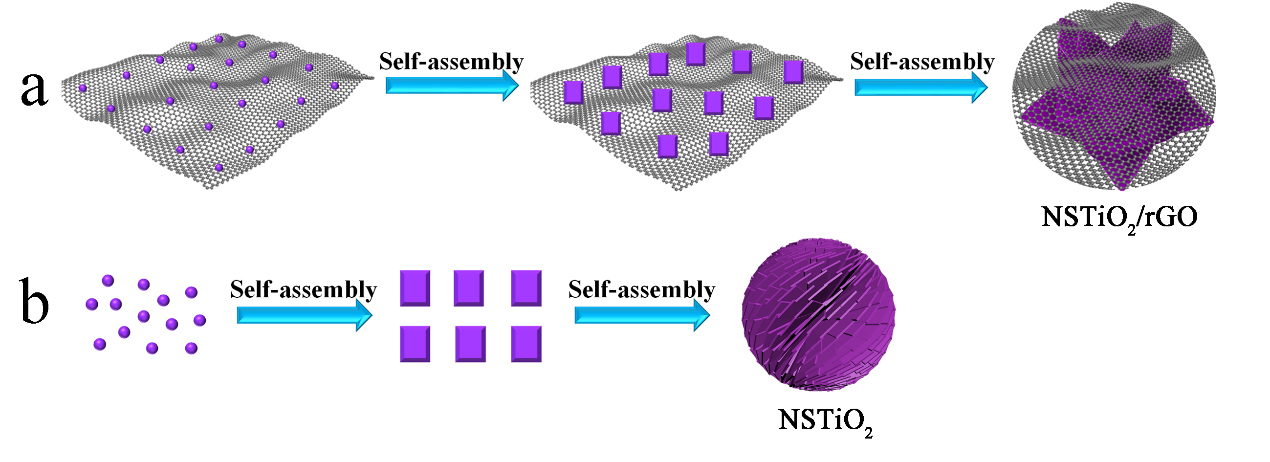


**Figure S1.** Synthesis and self-assembly processes of hierarchical (a) NSTiO_2_/rGO and (b) NSTiO_2_ micro/ nanostructures. The dots indicate newly formed pristine TiO_2_ nanocrystallites.


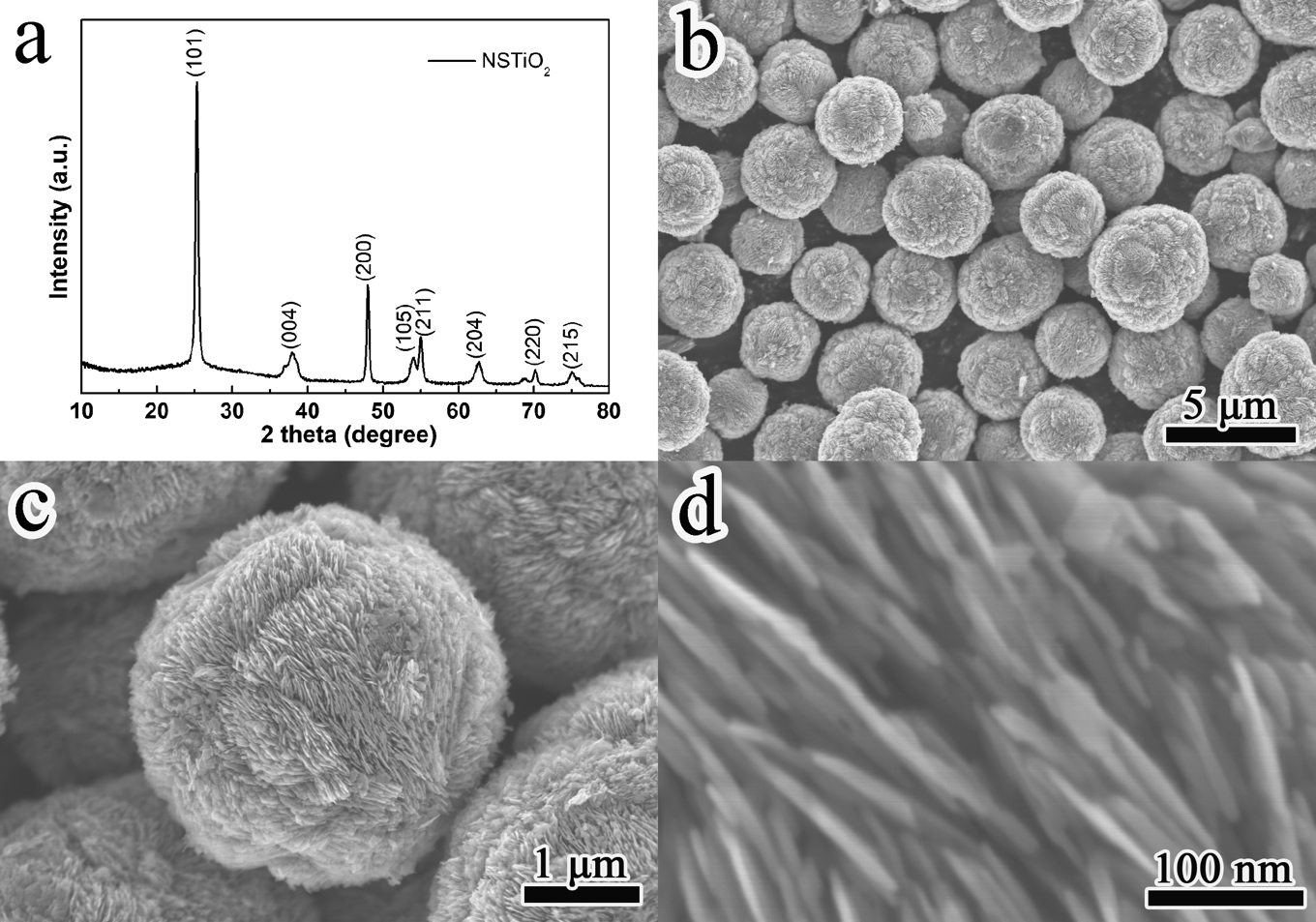


**Figure S2.** XRD pattern and SEM images of NSTiO_2_: (a) XRD pattern, (b)-(d) SEM images.


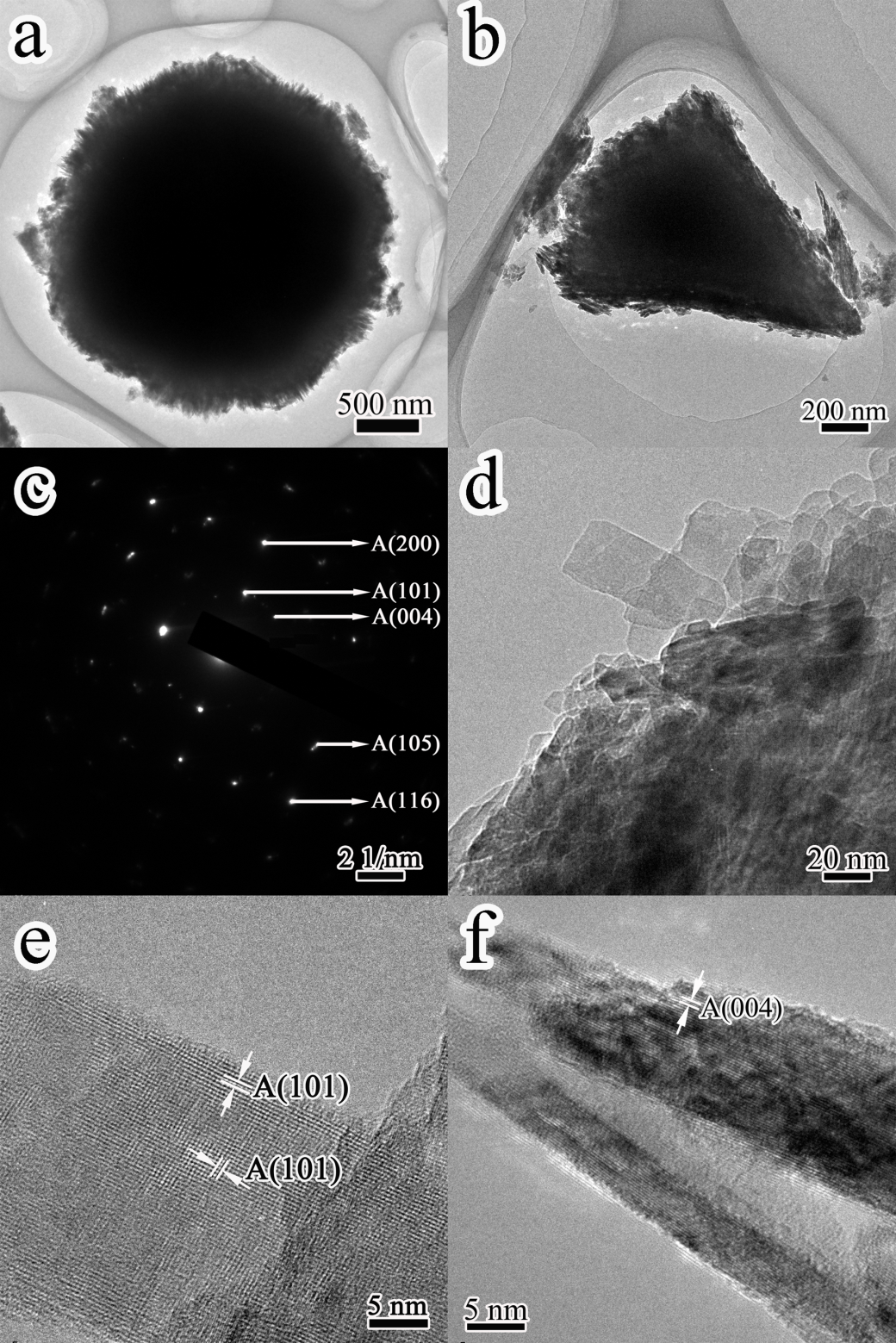


**Figure S3.** (a, b, d) TEM, (e, f) HRTEM and (c) SAED images of NSTiO_2_.


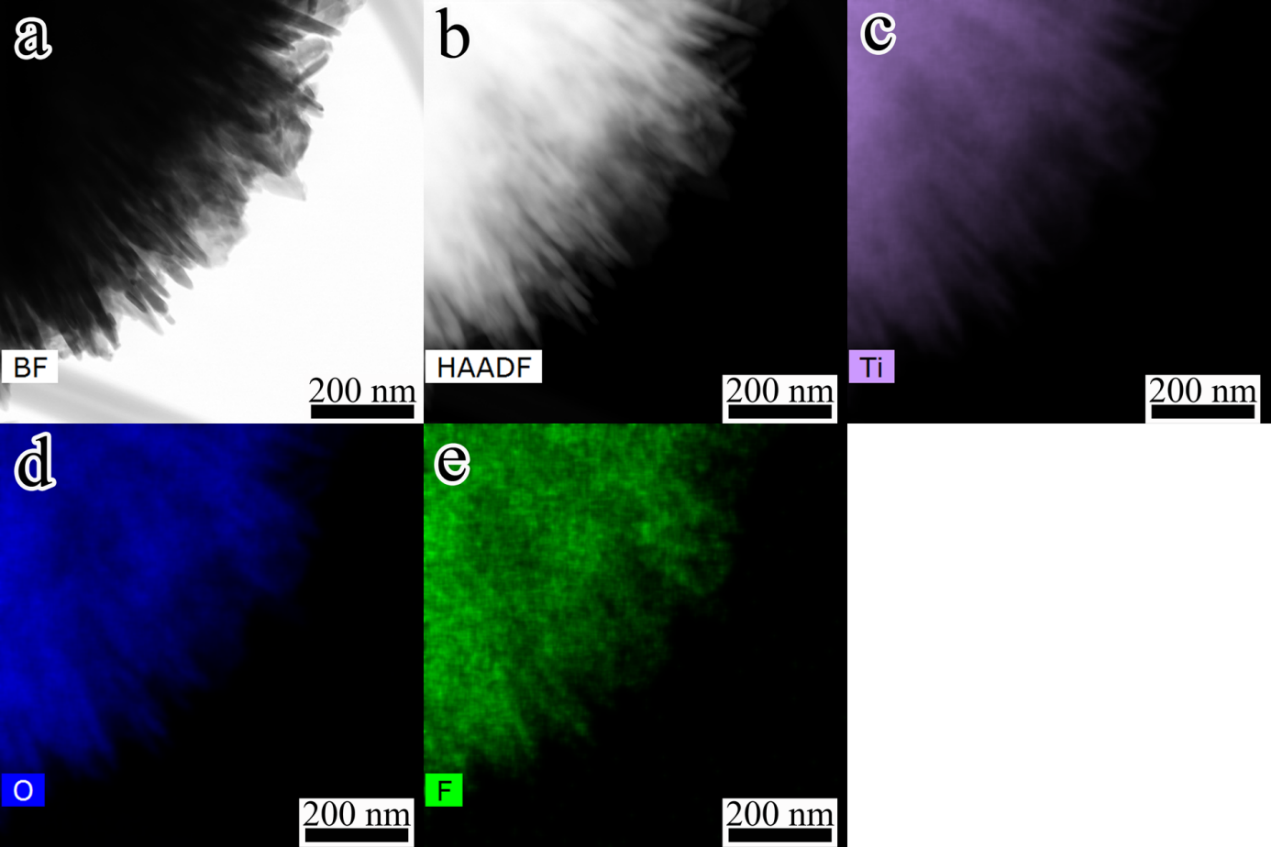


**Figure S4.** TEM characterizations of NSTiO_2_. (a) BF, (b) HADDF, (c)-(e) STEM-EDS mapping: (c) Ti; (d) O; (e) F elements.

**

**

**Figure S5.** XRD patterns of NSTiO_2_/rGO (blue curve), NSTiO_2_ (red curve), rGO (dark cyan curve) and GO (black curve).


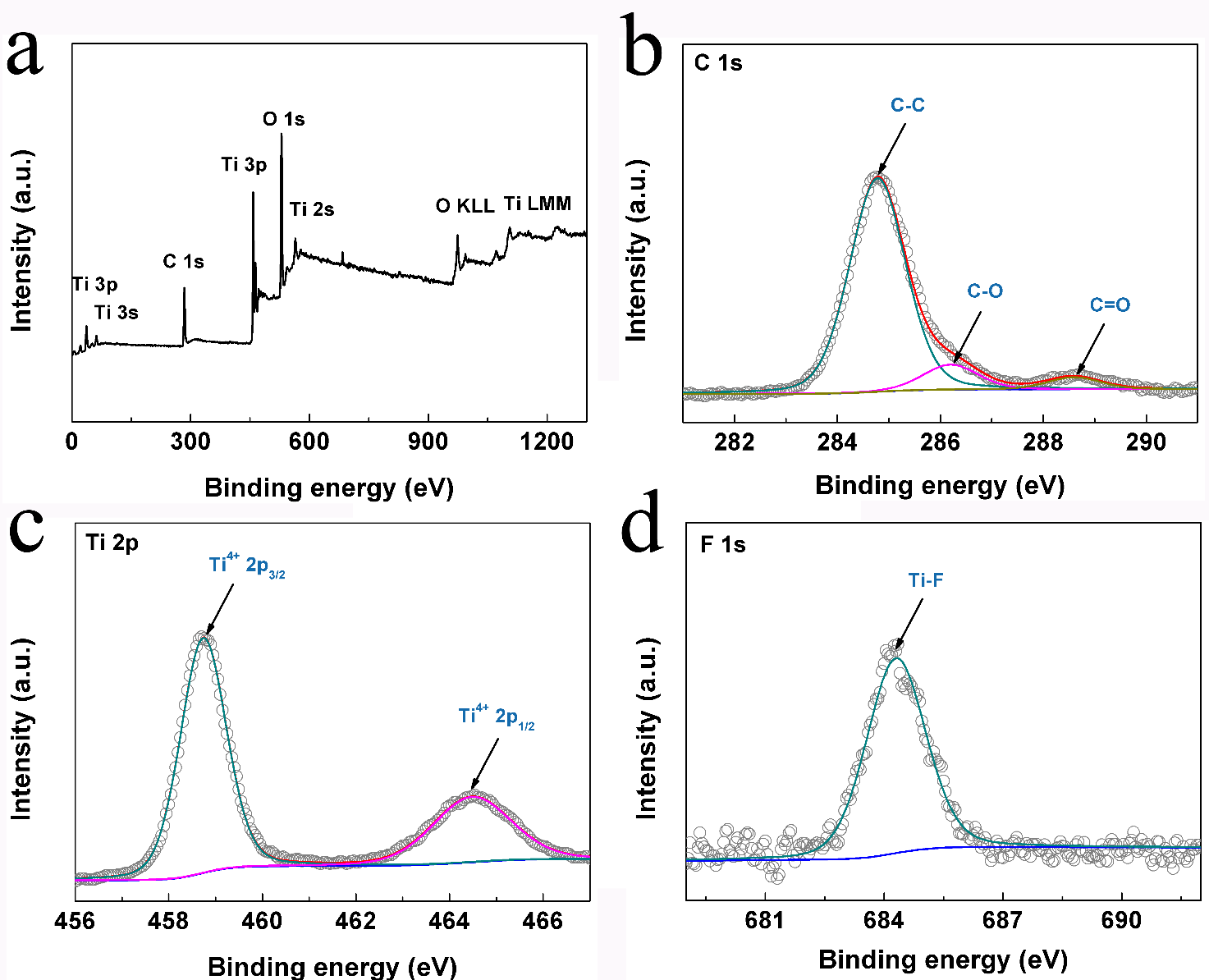


**Figure S6.** High-resolution XPS spectra of the NSTiO_2_/rGO : (a)XPS survey spectrum, (b)C 1s, (c) Ti 2p; (d) F 1s.


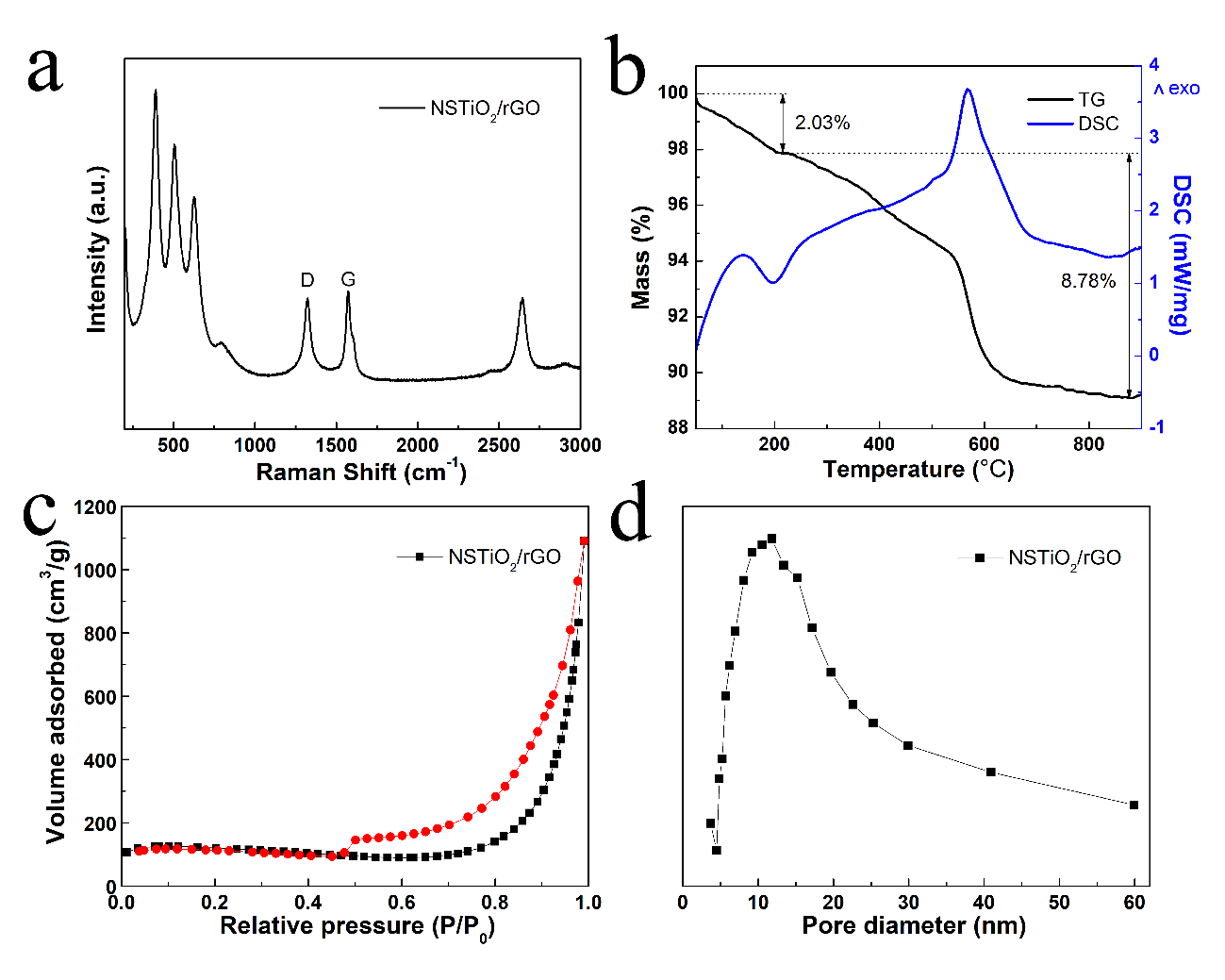


**Figure S7.** Various characterizations of NSTiO_2_/rGO. (a) Raman spectra, (b) TG and DSC curves, (c) N_2_ adsorption-desorption isotherms and (d) pore size distribution plots.


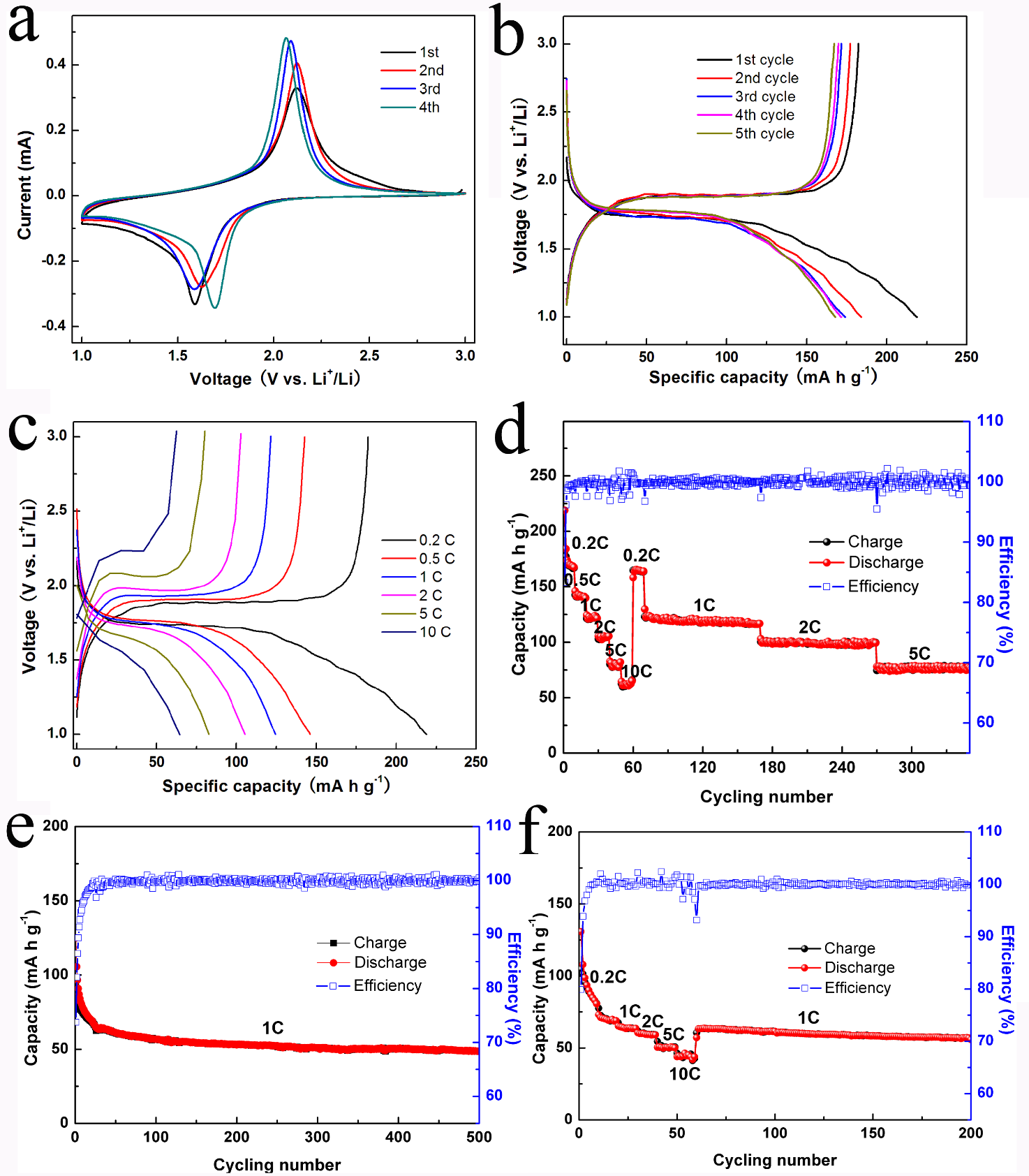


**Figure S8.** Electrochemical characterizations of the NSTiO_2_ and rGO anode materials: (a) representative CVs curves of NSTiO_2_ at a scan rate of 0.2 mV s^–1^, (b) discharge-charge profiles of NSTiO_2_ at 0.2 C, (c) discharge-charge profiles of NSTiO_2_ at various current densities, (d) rating and cycling performances of NSTiO_2_ at various current densities, (e) discharge-charge profiles of rGO at 0.2 C, (f) rating and cycling performances of rGO at various current densities.


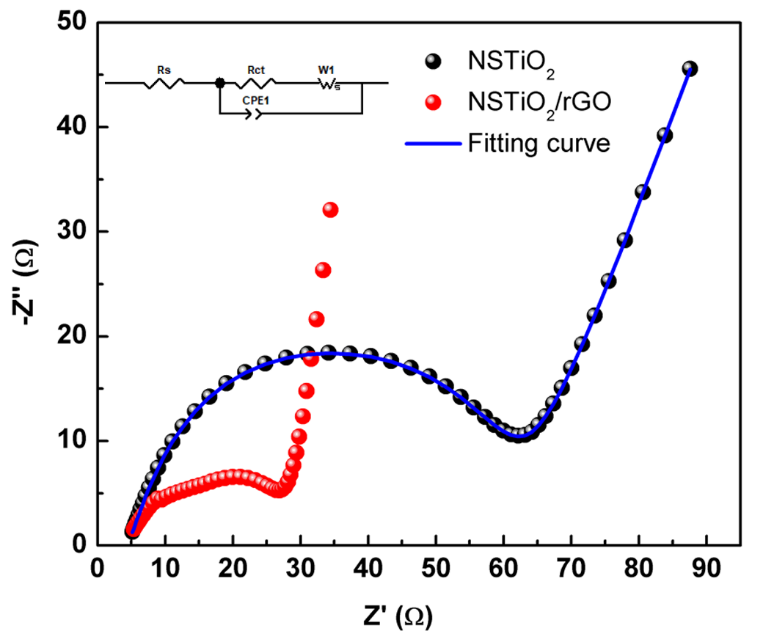


**Figure S9.** EIS spectra of NSTiO_2_ and NSTiO_2_/rGO (charged to 3.0 V) after 100 cycles at 1 C.


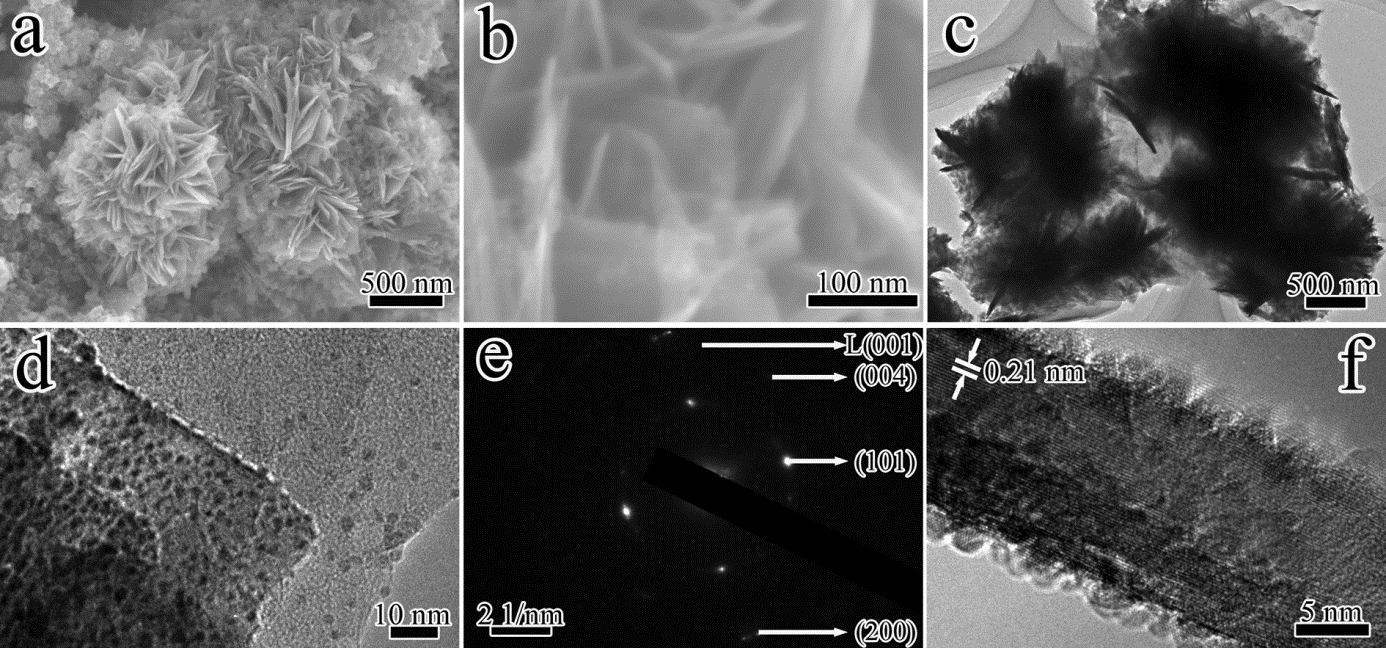


**Figure S10.** Microscopy images of the NSTiO_2_/rGO anode after 100 cycles at 1 C. (a-b) SEM image, (c, d, f) TEM images, (e) corresponding SAED image of the nanosheets in (d) and (e) HRTEM.


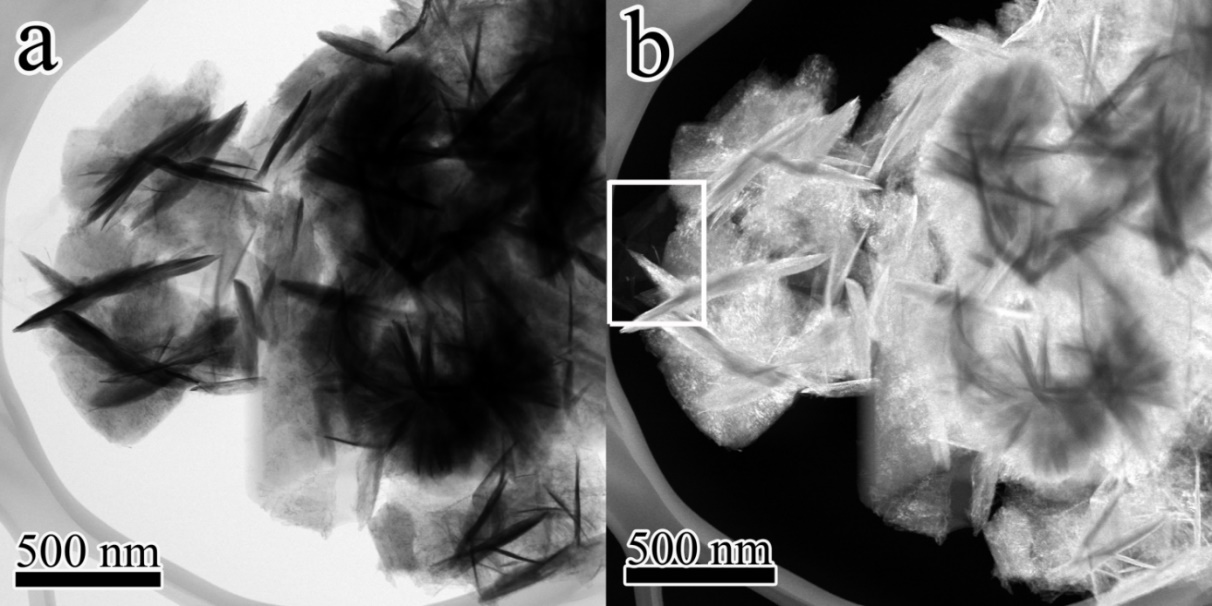


**Figure S11.** TEM characterizations of the NSTiO_2_/rGO anode material after 100 cycles at 1C: (a) bright field (BF) image and (b) dark field (DF) image. The DF image shows many nanoparticles on the surface of TiO_2_ nanosheets.


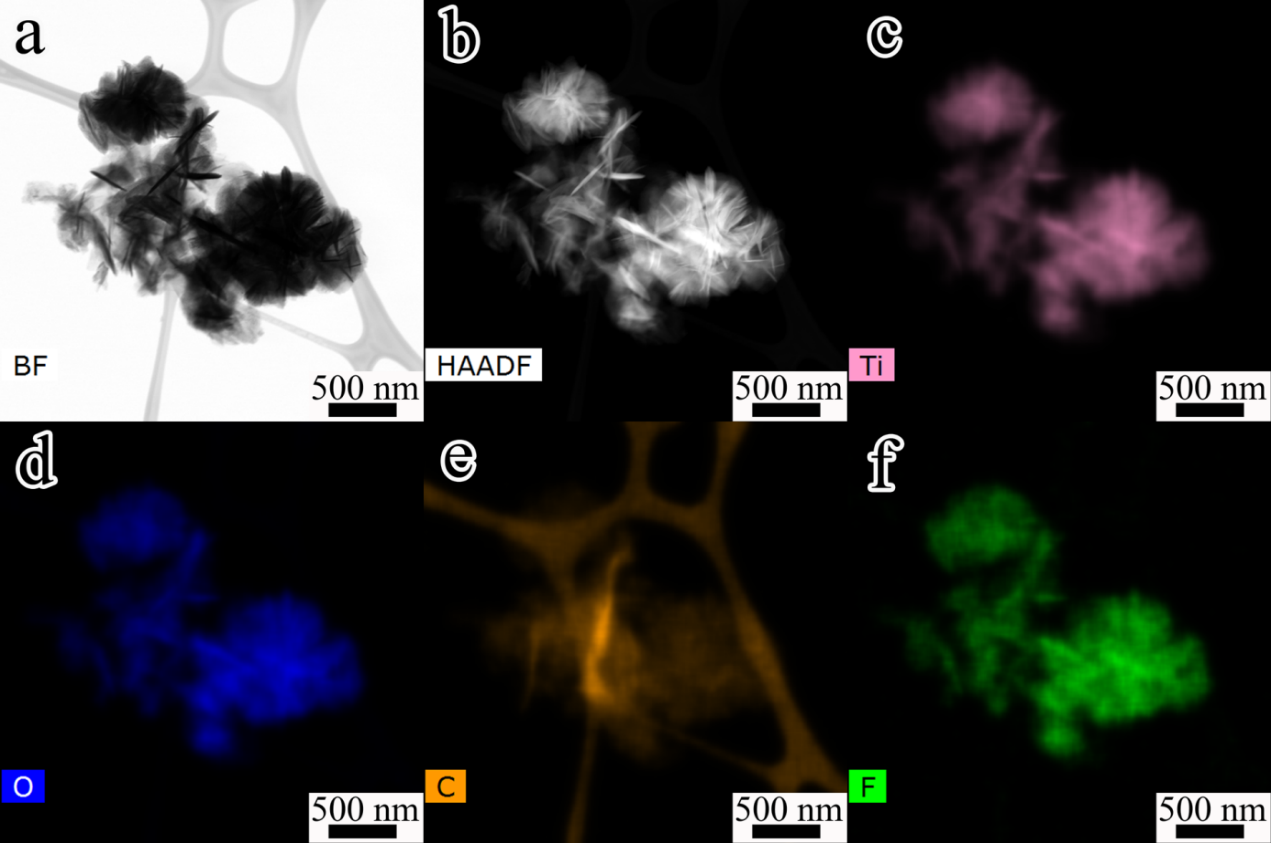


**Figure S12.** TEM characterizations of the NSTiO_2_/rGO anode material after 100 cycles at 1C: (a) BF, (b) HADDF, (c)-(f) STEM-EDS mapping: (c) Ti; (d) O; (e) C; (f) F.


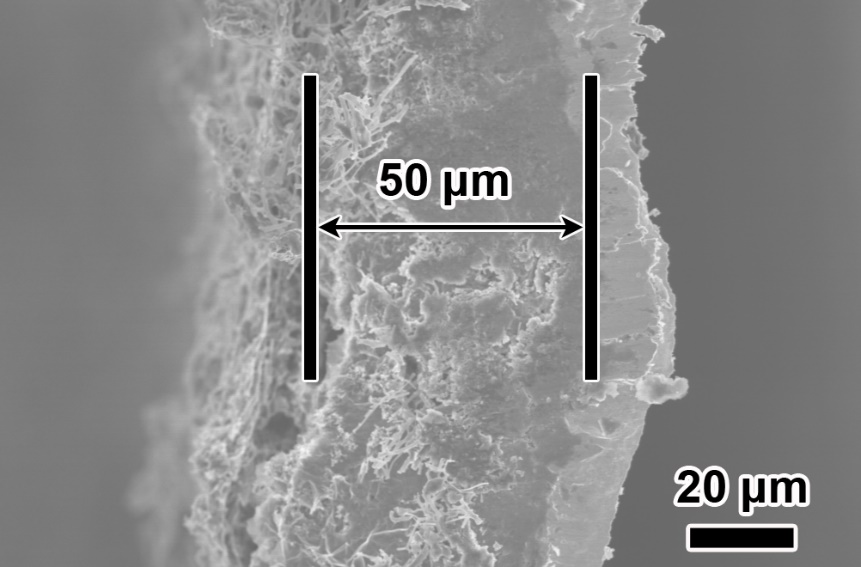


**Figure S13.** Electron microscopy characterization of the NSTiO_2_/rGO electrode.
